# Are X-rays the key to integrated computational materials engineering?

**DOI:** 10.1107/S205225251501951X

**Published:** 2015-10-21

**Authors:** Gene Ice

**Affiliations:** a Materials Science and Technology Division, Oak Ridge National Laboratory, PO Box 2008, Oak Ridge, TN 37831-6132, USA

**Keywords:** integrated computational materials engineering, nondestructive crystal structure mapping, stress tensor measurement

## Abstract

An **IUCrJ** paper by Levine *et al.* highlights the promise and challenge of 3D X-ray structural microscopy as a unique tool to test predictive models of materials behavior.

The ultimate dream of materials science is to predict materials behavior from composition and processing history. Owing to the growing power of computers, this long-time dream has recently found expression through worldwide excitement in a number of computation-based thrusts: integrated computational materials engineering, materials by design, computational materials design, three-dimensional materials physics and mesoscale physics. However, real materials have important crystallographic structures at multiple length scales, which *evolve* during processing and in service. Moreover, real materials properties can depend on the extreme tails in their structural and chemical distributions. This makes it critical to map structural distributions with sufficient resolution to resolve small structures and with sufficient statistics to capture the tails of distributions. For two-dimensional materials, there are high-resolution nondestructive probes of surface and near-surface structures with atomic or near-atomic resolution that can provide detailed structural, chemical and functional distributions over important length scales. However, there are no *nondestructive* three-dimensional probes with atomic resolution over the multiple length scales needed to understand most materials.

Although atomic resolution is still in the future, synchrotron-based X-ray microscopy is rapidly emerging as the standard for *nondestructive* three-dimensional characterization of materials structure at the macro-, meso- and nanoscales (Ice *et al.*, 2011 ▸). X-rays can penetrate tens to hundreds of micrometres into materials with spatial resolution approaching tens of nanometres. In addition, local lattice structure and strain measurements can be made at the part per ten thousand level, which is sufficient to distinguish inhomogeneous driving forces for materials evolution. During the last decade, a number of X-ray techniques have evolved to address the grand challenge of X-ray structural microscopy in three dimensions (Ice *et al.*, 2011 ▸; Clark *et al.*, 2015 ▸; Simons *et al.*, 2015 ▸).

As described in the paper ‘*Full elastic strain and stress tensor measurements from individual dislocation cells in copper through-Si vias*’ by Levine and co-workers (2015 ▸), an important advance toward this dream has recently been realised. In particular, the authors use their knowledge of the overall architecture of the copper vias to help untangle the complicated spatial distribution of the copper strain tensor in these technologically important materials. Their experiment utilizes specialized polychromatic microdiffraction tools including one-of-a-kind instrumentation for differential aperture microscopy on station 34-ID-E at the Advanced Photon Source (Levine *et al.*, 2015 ▸). By fully utilizing the existing instrumentation on station 34-ID-E and combining it with a knowledge of the device architecture, they have measured the full strain tensor at 7 well defined points with a good estimate of the uncertainties. Although the full strain tensor has previously been measured in a cross section for bent single-crystal silicon (Larson *et al.*, 2008 ▸), the Levine paper is the first time the full strain tensor has been measured in a case where the plastic and elastic strain tensors cannot be predicted from simple theory to help guide the measurements.

What makes this advance remarkable, is that this kind of measurement is precisely the grand challenge that station 34-ID-E was designed to address more than a decade ago. However, during the initial build out, instrumentation simply did not exist with the absolute precision, sensitivity and/or speed needed to map out meaningful three-dimensional volumes and the specially developed software was insufficient to deal with equipment imperfections. However, since its initial construction, a steady series of upgrades have accelerated data collection rates and improved precision on 34-ID-E. For example, full strain and/or dislocation tensor maps collected by Larson *et al.* (2008 ▸) captured roughly 1600 voxels while a more recent – and much faster measurements of local crystal orientations in deformed Al – mapped three-dimensional volumes with more than 300 000 micrometre-scale voxels (Fig. 1 ▸) (Budai, 2014 ▸).

Levine and colleagues have reminded us that the dream of full submicrometre strain tensor maps in three dimensions is indeed possible, even in challenging systems. What is needed now is a major effort to go beyond a demonstration on a particularly favorable system to make such measurements practical for virtually all systems. This will require large format detectors with unprecedented absolute position encoding and ultra-fast readout with single-photon counting statistics. It will also require optics and positioning instrumentation with unprecedented precision and speed. Moreover, special undulators are needed that harness the brilliance of new synchrotron magnetic lattice designs and optimize the X-ray beam properties for the unusual spectral requirements of polychromatic microdiffraction. It will also require heroic new software that can be calibrated to accommodate imperfections in the differential aperture and can efficiently utilize multiple and/or coded apertures to accelerate measurements.

Such an effort will not be cheap or quick. However, the payoff is an unprecedented capability: the ability to *nondestructively* map crystal structures and defects in three-dimensional volumes of arbitrary samples with resolutions of tens of nanometres within tens to hundreds of cubic micrometre volumes. Such a capability will provide powerful direct tests for computational models of materials structural evolution with point-to-point maps of evolving structure and defect distributions, including resolved strain maps. This kind of information is precisely what is needed to test emerging computational models and move us closer to the ultimate dream of predicting materials properties from composition and processing history (Ice *et al.*, 2013 ▸).

## Figures and Tables

**Figure 1:**
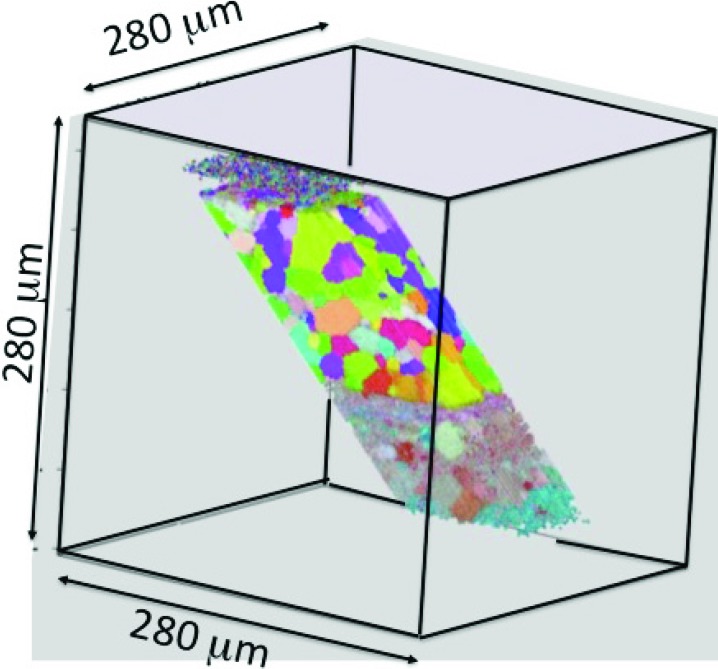
False color map showing grain orientations in an aluminium sample. The sample grain structure was measured before and after grain growth to test theoretical models of grain growth with 300 000 total voxels in each map. The measurements were sensitive to local crystallographic orientation but did not measure the strain tensor (Budai, 2014 ▸).
